# Transanal minimally invasive (TAMIS) mucosal resection with muscular plication for patients with obstructed defecation syndrome—A prospective pilot study

**DOI:** 10.1007/s10151-024-03101-3

**Published:** 2025-02-21

**Authors:** K. M. Widmann, C. Dawoud, D. Gidl, S. Riss

**Affiliations:** Department of General Surgery, Division of Visceral Surgery, Medical University Vienna, Waehringer Guertel 18-20, 1090 Vienna, Austria

**Keywords:** Obstructed defecation syndrome, TAMIS mucosectomy, Rectocele, Intussusception, Internal Delorme’s procedure

## Abstract

**Background:**

Rectocele and intussusception are frequently observed during defecography as potential contributors to obstructed defecation syndrome (ODS). We aimed to describe our initial experience with transanal minimally invasive surgery (TAMIS) mucosectomy with muscular plication, as a novel surgical approach to treat patients with ODS.

**Methods:**

Conducted between August 2021 and October 2023 at the Medical University of Vienna, 11 patients (8 female) were prospectively enrolled and underwent TAMIS mucosectomy with circular mucosectomy and longitudinal muscular plication (internal Delorme’s procedure). Functional outcome and quality of life were assessed by using standardized questionnaires pre- and postoperatively. The median follow up time was 16 months.

**Results:**

In defecography rectal intussusception could be observed in all patients and rectocele was found in nine patients (81.8%). The median age at the time of surgical procedure was 56 years (range 28–76 years). Neither intraoperative nor postoperative complications occurred. The median ODS score decreased from 16 to 11 points (*p* = 0.171), and four out of five patients (80%) with preexistent fecal incontinence reported improvement of their symptoms postoperatively (80%), though one patient had new onset of fecal incontinence symptoms. No significant changes could be demonstrated in terms of quality life by using the Short-Form Health Survey 12 (SF-12) survey.

**Conclusions:**

Our initial results showed that TAMIS mucosectomy is a safe technique, offering a viable alternative transanal approach for treating symptomatic ODS. Future studies with a larger sample size and a longer follow-up period should enhance the robustness of our preliminary findings.

## Introduction

Obstructed defecation syndrome (ODS) is a specific type of constipation that can be characterized by several symptoms: hard, pellet-like stools, a persistent feeling of incomplete evacuation, excessive straining during bowel movements, urgency, and the need for digital assistance [1, 2].

The underlying causes of ODS are likely to be complex and involve a combination of functional and anatomical factors that disrupt the regular process of defecation. Among these, rectocele and rectal intussusception are the most prevalent anatomical abnormalities associated with ODS that can be visualized in dynamic defecography [3].

Rectoceles are characterized by the herniation of the rectal wall, either anterior bulging toward the vagina or posterior toward the sacrum [4]. Rectal intussusception or internal rectal prolapse describes the protrusion of the rectal wall into the lumen without reaching the anus. Patients with intussusception may not only suffer from obstructed defecation, but also fecal incontinence [5]. However, it is worth noting that a considerable number of patients have anatomical changes but do not experience obstruction during defecation. Therefore, treatment options are dependent on the causes and the severity of symptoms. After exploring conservative options, surgery is considered a potential next step in well-selected patients. [6].

Various surgical approaches for treating ODS have been described in literature [2, 7]. Minimal invasive ventral mesh rectopexy has gained increased popularity among surgeons in the last decade, revealing moderate-to-good results in published literature [8, 9]. Notably, transanal procedures such as stapled transanal resection of the rectum (STARR) were also commonly conducted previously, with promising success rates in several studies [10]. However, various complications, including fecal incontinence and stool urgency, potentially partly related to the staple line, have been reported. Thus, its widespread use has been reduced significantly. [11].

Conventional circumferential sutured transanal mucosectomy with plication has been proposed recently for treating an internal rectal prolaps [12]. We hereby report our first experience with the performance of a non-stapled mucosal resection by transanal minimally invasive surgery (TAMIS internal Delorme’s procedure). Therefore, our study was designed to evaluate the safety and short-term outcomes of this novel approach.

## Materials and methods

Conducted at the Medical University of Vienna between August 2021 and October 2023, this single-arm exploratory pilot study examined the efficacy and safety of transanal minimally invasive surgery in patients diagnosed with obstructed defecation syndrome (ODS). Ethical approval was granted by the Ethics Committee of the Medical University of Vienna (#1415/2022).

Patients eligible for transanal minimally invasive surgery had an identified organic cause for ODS, specifically symptomatic intussusception with or without rectocele. Confirmation through medical examinations included routinely performed defecography in our department. All patients had undergone unsuccessful conservative treatments over a period exceeding 6 months. Conservative therapy involved lifestyle changes such as increased physical activity, a fiber-rich diet, proper hydration, and stool regulation with suppositories or oral laxatives.

Demographic data and comprehensive medical histories, including anorectal function and defecography results, were systematically recorded. Additionally, information on functional outcome was obtained through the administration of standardized questionnaires at baseline and at follow-up assessments. To assess constipation, we used the Obstructive Defecation Syndrome-Score (ODS-S), and for measuring fecal incontinence symptoms, the St. Mark’s Incontinece Score (SMIS) was utilized. The Short-Form Health Survey 12 (SF-12) was used to assess the quality of life.

### Preoperative defecography

Defecography was performed routinely in patients with persistent obstructed defecation symptoms to exclude morphological causes. The examination was conducted in a radiology department and interpreted by certified radiologists.

Rectocele size was assessed using a standardized technique, measuring the distance from the anterior-most point of the anterior rectal wall to an extrapolated line representing the normal position of the rectal wall. Then, it was categorized on the basis of the depth of its protrusion: small (< 2 cm), moderate (2–4 cm), or large (> 4 cm) [13].

The presence and severity of an intussusception was classified according to the Oxford Rectal Prolapse Grade from grade I at the level of the rectocele to grade IV descending into the anal canal. Grade V presented an external rectal prolapse [14].

### Surgical procedure

**Fig. 1:**
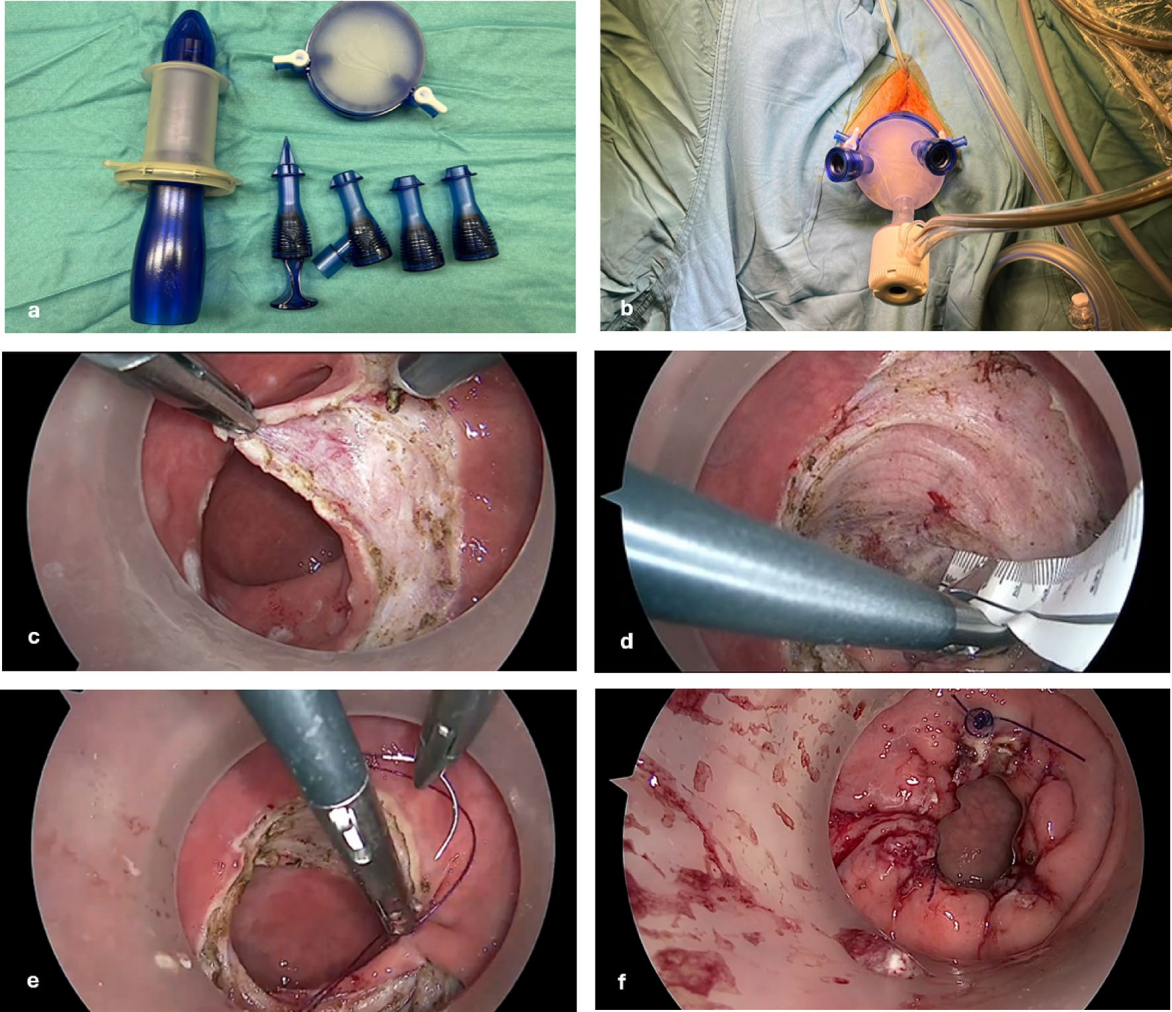
Technical steps of TAMIS mucosectomy [15]: **a** and **b**: trocars (two 5 mm trocars and one 10 mm trocar) and the inserted gel port, **c** mucosal excision starting at three o’clock, **d** 360° circular mucosal excision of 5 cm, **e** longitudinal muscular plication using 3.0 V-Loc suture, **f** securing the closure using 2.0 PDS sutures

Patients were hospitalized and received a mechanical bowel preparation 1 day prior to surgery. Perioperative single-shot antibiotic prophylaxis consisted of intravenously given metronidazol 1.5 g and cefuroxime 1.5 g. The procedure was performed with patients under general anesthesia in lithotomy position. After anal inspection and inserting the gel port (Fig. 1a, b; GelPOINT® Path Transanal Access Platform, Applied Medical, Rancho Santa Margarita, CA) pneumorectum was established with CO_2_ insufflation. A marking of the excision line was done using diathermy starting at three o’clock 2–3 cm above the dentate line (Fig. 1c). Subsequently, a 360° circular mucosal excision of around 5 cm followed without penetration of muscular layers (Fig. 1d). Finally, longitudinal muscular plication was conducted using a 3.0 V-Loc (Covidien V-Loc™, Covidien, Mansfield, MA) suture similarly to Delorme procedure (Fig. 1e). The closing was further secured using 2–3 2.0 PDS suture knots with extra corporal knot tying (Ethicon PDS™, Johnson & Johnson, Sommerville, NJ, USA) (Fig. 1f). A video vignette performing TAMIS mucosectomy in a patient with ODS has recently been published by our research group [15].

### Outcome measurements

The effectiveness of the intervention was evaluated by using the ODS-Score as the primary outcome. The ODS-S, introduced by Altomare et al., consisted of eight items with four to five response options, each assigned a score of zero (no symptoms) to three or four points (severe symptoms). The overall ODS score was calculated by summing the individual item scores, with a maximum possible score of 31 [16]. Furthermore, changes on fecal incontinence symptoms were assessed by using the St. Mark’s Incontinence Score (SMIS). The SMIS score can reach a maximum of 24 points, depending on the severity of fecal incontinence [17]. To assess quality of life, we used the Short Form Health Survey – 12 (SF-12), the shortened version of the original SF-36. The questionnaire consisted of a mental and physical component. The individual categories were summed and converted into T-scores. A T-score of 50 corresponded to the average in the general population, and differences of 5 or more units were considered clinically significant [18].

### Statistical analysis

Ordinal data were described as medians and ranges. Metric data were summarized as mean and standard deviation, when skewed distribution data were also described as medians and ranges. Qualitative data were described with counts and percentages.

For analyzing the endpoints comparing pre- and postoperative measurements, the Wilcoxon signed rank test was used for continuous values. For comparing categorical variables, Fisher’s exact test was used. A *p*-value < 0.05 was considered statistically significant. All statistical data analysis was conducted using SPSS (IBM SPSS Statistics for Mac, Version 29.0)

## Results

### Patient characteristics

From August 2021 to October 2023, 11 patients (8 female, 3 male) were prospectively enrolled in this study and underwent transanal minimally invasive surgery (TAMIS) mucosectomy. All patients had obstructed defecation and five patients (45.5%) had mixed obstructed defecation and fecal incontinence symptoms. Demographic data are listed in Table 1.

**Table 1:** Demographic data of the included patients

Demographics	
Age (years); median (range)	56 (28–76)
Female sex, *n* (%)	8 (72.7)
BMI (kg/m^2^)	26.6 (17.6–35.7)
Clinical history	
History of smoking, *n* (%)	2 (18.2)
Childbirth, *n* (%)	6 (54.5)
Previous pelvic floor surgery, *n* (%)	7 (63.6)
Rectopexy, *n* (%)	4 (36.4)
STARR, *n* (%)	1 (9.1)
Hysterectomy, *n* (%)	1 (9.1)
Colporrhaphy, *n*(%)	1 (9.1)
Hemorrhoidectomy, *n* (%)	3 (36.4)
Fistulotomy, *n* (%)	1 (9.1)

*BMI* body mass index

### Preoperative defecography

In defecography, a rectal intussusception was observed in all patients. Additionally, nine patients (81.8%) had a rectocele [small (*n* = 1,11.1%), moderate *n* = 4, 44.4%, large *n* = 3, 33.3%)]. Detailed defecography results are described in Table 2.

**Table 2:** Defecography findings of included patients

Rectocele, *n* (%)	9 (81.8)
Localization; *n* (%)	
Anterior	4 (36.4)
Posterior	0
Anterior and posterior	5 (45.5)
Size of rectocele in mm, median (range)	
Anterior	40 (13–50)
Posterior	25 (17–30)
Intussusception, *n* (%)	11 (100)
Localization, *n* (%)	
Proximal third of the rectum	0
Middle third of the rectum	1 (9.1)
Distal third of the rectum	3 (27.3)
Reaching up to the anal canal	3 (27.3)
Intraanal	4 (36.4)
Oxford Grading (I–V), *n* (%)	
I	0
II	4 (36.4)
III	3 (27.3)
IV	4 (36.4)
V	0

### Surgical outcome

The median operation time was 79 min (range 50–135 min). The median resection line was 50 mm (range 40–65 mm). There were no intraoperative or early postoperative complications during the patients’ hospitalization. The median hospital stay was 3 days (range 2–6 days). There were two patients who stayed longer than the average: one, who stayed for 6 days, had subjective dyspnea 2 days postoperatively. A spiral computed tomography (CT) was performed to rule out a pulmonary embolism, which was completely unremarkable. The patient’s symptoms disappeared on the same day. The other, who stayed for 5 days, remained for pain management upon request. No postoperative complications were reported in this case either. In total, no postoperative major morbidity or mortality could be observed during the follow-up time.

### Functional outcome

**Fig. 2:**
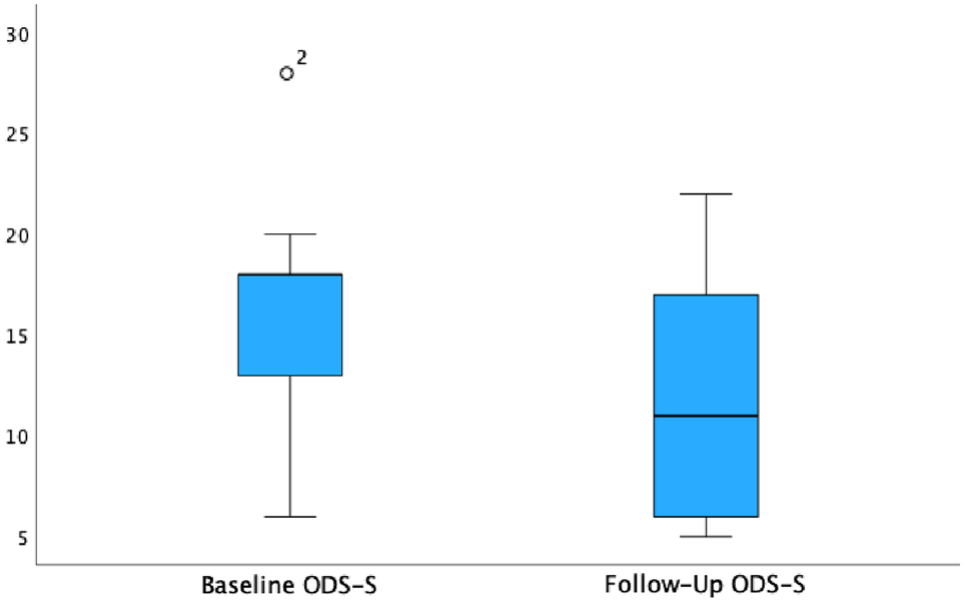
Obstructed defecation syndrome score (ODS-S): the median ODS-S decreased from 16 to 11 points (*p* = 0.171)

**Fig. 3:**
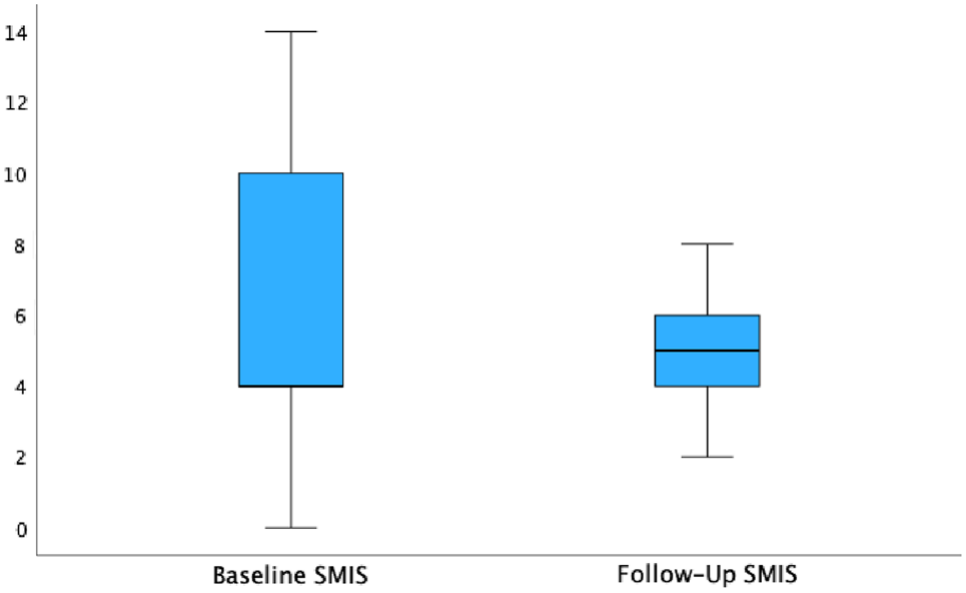
The median St. Mark’s Incontinence Score was lower postoperatively (6 versus 5 points)

**Fig. 4:**
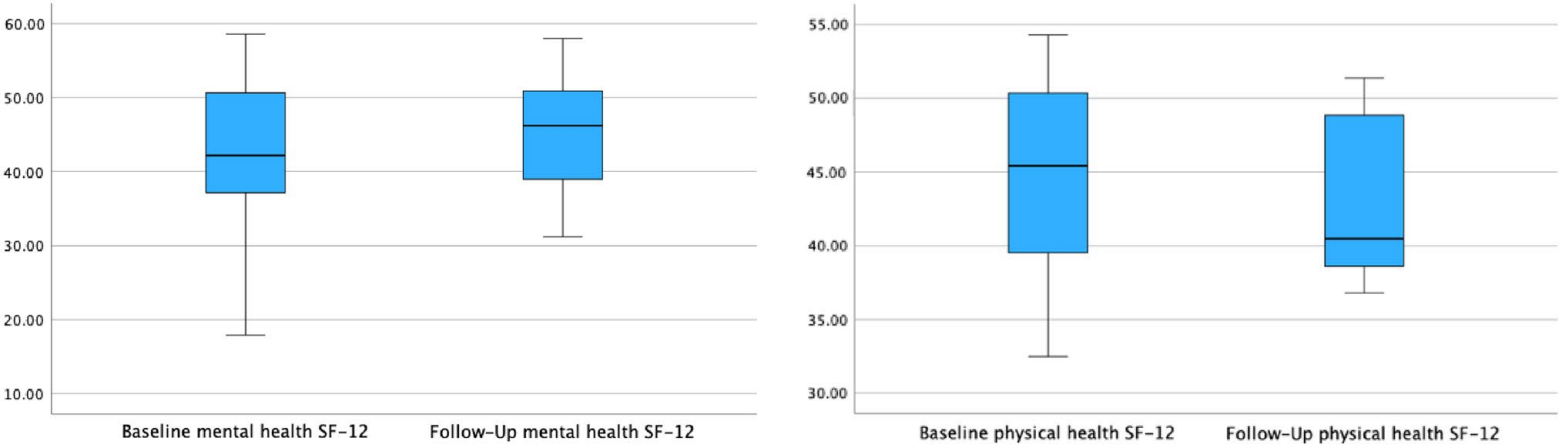
Baseline and follow-up: mental and physical health SF-12 health survey

The median follow-up time was 16 months (range 2–26 months). The median ODS score was lower postoperatively (11 points) compared with preoperatively (16 points) (*p* = 0.171, Fig. 2). A decrease of the median St. Mark’s Incontinence Score from 6 to 5 points could be observed without reaching statistical significance (*p* = 0.498, Fig. 3). No significant changes were observed in the SF-12, nonetheless a mild median increase of the mental component could be demonstrated (see Table 3 and Fig. 4).

**Table 3:** Quality of life and outcome after operation

	Baseline (*n* = 10)	Follow-up (*n* = 9)	Wilcoxon signed rank test, *p*
SF-12			
Physical health, median (range)	44.65 (32.48–54.30)	40.48 (36.81–51.37)	0.441
Mental health, median (range)	41.60 (17.89–58.62)	46.20 (31.24–58.00)	0.767
ODS-S, median (range)	16 (6–28)	11 (5–22)	0.171
SMIS, median (range)	6 (0–15)	5 (2–8)	0.498

*SF-12* Short Form health survey 12, *ODS-S* obstructed defecation syndrome score, *SMIS* St. Mark’s Incontinence Score

### Obstructed defecation symptoms (ODS-S)

Among the nine patients who completed the ODS-S in the follow-up, a reduction in defecation symptoms was observed in six (66.7%), while three (33.3%) continued to experience persistent symptoms. All categories of the questionnaire were less frequently reported in the subsequent evaluation. Particularly, a statistically significant reduction was achieved in the use of enemas and suppositories (*p* = 0.042) (see Table 4).

**Table 4:** Baseline versus follow-up in obstructed defecation symptoms

Obstructed defecation symptoms	Baseline (*n* = 10)	Follow-up (*n* = 9)
Time for defecation > 10 min, *n* (%)	6 (60)	4 (44.4)
Feeling of incomplete evacuation ≥ 1×/week, *n* (%)	9 (90)	6 (66.7)
Strong squeezing in defecation ≥ 50% of the time, *n* (%)	6 (60)	3 (33.3)
> 5 attempts to defecate per day; *n* (%)	4 (40)	0
Using laxatives ≥ 1×/week	4 (40)	3 (33.3)
Using enemas ≥ 1×/week	6 (60)	3 (33.3)
Anal/vaginal digitation ≥ 1×/week	5 (50)	3 (33.3)
Stool consistency		
Soft	4 (40)	6 (66.7)
Hard	4 (40)	0
Hard and few	1 (10)	0
Fecaloma formation	1 (10)	3 (33.3)

### Fecal incontinence symptoms (SMIS)

At follow-up, four out of five patients (80%) with moderate fecal incontinence reported less fecal incontinence symptoms after the operation with a median reduction from 6 to 5 points in SMIS. None of them reached full continence. All five experienced episodes of fecal incontinence with liquid stool preoperatively—three had episodes once a month, while two experienced them several times a month. Additionally, these two patients also had stool loss involving solid stool, which has been resolved in at least one patient after the operation.

In the follow-up period, an individual who had not previously experienced fecal incontinence reported the onset of new symptoms. The symptoms were self-limiting after 6 months.

### Previous and secondary interventions for treating obstructive defecation

Of the 11 patients in our cohort, 9 had a history of at least one prior pelvic floor surgery before undergoing TAMIS mucosectomy, 4 had previously undergone a ventral mesh rectopexy, and 1 of these 4 patients had also undergone a STARR procedure.

During the postoperative observation period, two patients underwent additional surgery for the treatment of obstructive defecation syndrome. One patient received a Re-TAMIS mucosectomy after 14 months, while the other patient underwent laparoscopic ventral mesh rectopexy after 7 months.

## Discussion

The purpose of our study was to evaluate the safety and short-term outcomes of TAMIS mucosectomy with longitudinally muscular plication in patients with ODS associated internal rectal prolaps. A similar operation has been reported by Cao et al., without using a minimally invasive platform [12]. Thus, to the authors’ knowledge, our study represents a novel technical approach for treating ODS.

In general, TAMIS was first described in 2010 by Atallah, Albert, and Larach as a fusion of single-incision laparoscopic surgery and transanal endoscopic microsurgery (TEM) [19]. Though the primary aim was to provide access to the upper and middle third of the rectum for the removal of benign and early stage malignant lesions, TAMIS has demonstrated versatility in various medical scenarios, including fistula repair, management of bleeding, and assisting in total mesorectal excision for rectal cancer [20]. In this context, the idea emerged to utilize the device for the treatment of ODS with accompanying structural abnormalities as an alternative to other transanal approaches, especially the stapled transanal rectal resection (STARR).

In accordance with our results, TAMIS has also been proven to be a safe and low-complication procedure for removing various rectal lesions. Kang et al. reported a minor complication rate of 13.5% including diarrhea and fecal incontinence postoperatively, which only lasted for up to 1 week. No patients experienced any dehiscent or stricturing issues at the site of the surgical repair [21]. Similar results were obtained by Quaresima et al. with a complication rate of around 9.6%, which could be resolved without any reoperations or invasive treatments [22].

The most common alternative transanal approach for managing ODS, the STARR procedure, involves staplers for a mucosal (including muscle fibers) resection compared with TAMIS as a non-stapling procedure [23]. STARR initially showed promising outcomes in alleviating ODS, yet demonstrated a subsequent decline over time [11]. Lenisa et al. reported an improvement of ODS in 77.3% after 12 months, while Zhang et al. reported similar results with improved constipation symptoms in 90% [3, 24]. Compared with STARR, we could demonstrate a decline of symptoms in about 70%, without reaching a statistical significance, most likely due to our small sample size.

Although the European STARR registry highlighted a notable success rate for addressing ODS, it is crucial to acknowledge the associated challenges, since the procedure is accompanied by a substantial complication rate of up to 36%. The most common complications included an increased sense of urgency during bowel movements (20.0%), followed by prolonged discomfort (7.1%), difficulty in emptying the bladder (6.9%), instances of bleeding (5.0%), infections of the anal and rectal area (4.4%), complications related to the stapling process (3.5%), and de novo fecal incontinence (1.8%) [25]. With the TAMIS mucosectomy, we hoped to overcome some of those limitations. Using absorbable sutures has the potential advantage of avoiding fecal urgency, as it commonly occurred in patients after STARR procedure. Urgency symptoms were observed in the majority of our patients as well, but resolved completely in all participants. Additionally, there was one patient with a new onset of fecal incontinence, which resolved after 6 months.

In our postoperative assessments we observed changes in bowel movements, with a higher numbers of patients reporting soft stool after surgery. Interestingly, the formation of fecalomas increased from one to three patients. The increase in fecaloma formation could reflect a temporary change in motility postoperatively but needs to be investigated in future studies.

We compromised four patients who had previously undergone laparosopic ventral mesh rectopexy prior to TAMIS mucosectomy. In literature, laparoscopic ventral mesh rectopexy demonstrated low long-term recurrence rates and a favorable profile regarding de novo constipation [26]. In comparison, TAMIS offers a mesh-free, minimally invasive alternative with promising initial outcomes but still limited long-term data on recurrence. In our observation period, two patients required reoperation for recurrence: one underwent Re-TAMIS mucosectomy, and the other laparoscopic ventral mesh rectopexy. Data on Re-Do ventral rectopexy are scarce but represent another option for recurrent disease [27]. Currently, there is no clear evidence to suggest which surgical approach is superior for managing recurrences.

Due to several cases of newly developed fecal incontinence after STARR operation, a strict indication was applied in patients with already preexisting sphincter weakness [2]. According to the European consensus guidelines on the surgical management for ODS, it was recommended to favor an abdominal approach in those patients instead [28]. Thus, laparoscopic ventral mesh rectopexy represented the preferred technique thus far, with significant improvement in constipation ranging from 52% to 84%. Although it is considered as a safe technique, there are also some complications to consider that rarely occurred [9]. Adverse events relating to the mesh have been especially discussed, particularly noting erosion rates in 2% to 4%. Suggestions have been made that utilizing biological grafts and absorbable stitches may reduce these associated risks [8]. Furthermore, extensive practice for precise tack placement when fixing the mesh is required. This is crucial to avoid damaging blood vessels and nerves, preventing bleeding during surgery, and chronic pelvic pain in the long term [29].

It is assumed that TAMIS could potentially cause fewer anal sphincter impairments, because of the flexibility, softness, and small size (30 mm diameter) of the TAMIS port, which may facilitate a safe and gentle transanal access [30]. This is supported by the findings of Schiphorst et al., which showed that preoperative incontinence even improved in 88% of patients after TAMIS surgery, with only two patients experiencing a deterioration of preexisting symptoms [31]. Similarly, Verseveld et al. demonstrated a 79% improvement rate of fecal incontinence, although five patients showed a worsening of symptoms. This was argued to be exclusively associated with patients in which the resected lesion was located very distally [32]. However, it is essential to note that a direct comparison is not entirely feasible, since TAMIS was utilized here for rectal tumors and not for treating rectal intussusception or rectocele. In our patient cohort, a mild improvement in incontinence symptoms was observed in four out of five patients (80%), with one patient complaining about newly developed moderate fecal incontinence.

Despite our study providing initial valuable insights into the use of TAMIS mucosectomy for ODS, there are some limitations that need to be acknowledged. A drawback of our study was the small sample size, comprising only 11 individuals. Furthermore, the indication for TAMIS mucosectomy still needs to be defined in future studies, but may be comparable to STARR procedure. In the present pilot study, we included primarily patients who were not ideal candidates for rectopexy. For example, we enrolled patients who had multiple laparotomies previously and younger patients in whom we wanted to avoid to implant a synthetic mesh. Patients with recurrent disease after previous ventral rectopexy may also be suitable candidates, as four such patients achieved good results after surgery. Whether this procedure is similarly effective in comparison with the commonly conducted ventral rectopexy still needs to be defined. Our initial results in both primary and recurrent cases suggest that TAMIS mucosectomy is a safe and potentially effective alternative, but future studies with a larger sample size and a longer follow-up period should enhance the robustness of our preliminary findings.

## Conclusions

Our initial results revealed that TAMIS mucosectomy with muscular plication is a safe technique, offering a viable alternative anal approach for treating ODS related to rectocele and intussusception.

## Data Availability

The raw datasets generated during and/or analyzed during the current study are not publicly available due to the sensitive nature of the questions asked in this study but are available from the corresponding author at reasonable request.
